# The protective effect of vitamin C on phenylhydrazine-induced hemolytic anemia on sperm quality and in-vitro embryo development in mice

**DOI:** 10.18502/ijrm.v16i12.3685

**Published:** 2019-01-28

**Authors:** Mojtaba Karimipour, Zahra Dibayi, Abass Ahmadi, Masoumeh Zirak Javanmard, Elnaz Hosseinalipour

**Affiliations:** ^1^Department of Anatomy and Histology, Faculty of Medicine, Urmia University of Medical Sciences, Urmia, Iran.; ^2^Department of Basic Sciences, Faculty of Veterinary Medicine, Urmia University, Urmia, Iran.

**Keywords:** *Vitamin C*, * Phenylhydrazine*, * Sperm*, * Fertilization*, * Mice.*

## Abstract

**Background:**

Phenylhydrazine (PHZ) induced anemia and was shown to have harmful effects on the male reproductive system.

**Objective:**

To investigate the protective effect of vitamin C (Vit C) on sperm parameters quality, in vitro fertilization potential and embryonic development in a mouse model of hemolytic anemia induced by PHZ.

**Materials and Methods:**

Thirty-two NMRI adult male mice (*n* = 8/each) were randomly classified into four groups. Group I (control) received normal saline, Group II (PHZ) received 8 mg/100 gr body weight PHZ as initial dose, continued by 6 mg/100 gr intraperitoneally every 48 hr, Group III (Vit C) received Vit C (10 mg/kg, daily, intraperitoneally), and group IV (PHZ + Vit C) received PHZ and Vit C. After 35 days, sperm quality parameters, the percentage of sperm with DNA damage and in vitro fertilization outcomes up to blastocyst stage were evaluated.

**Results:**

A significant (*p* < 0.001) reduction in all of the sperm parameters (count, motility, viability and normal morphology) were observed in group II (PHZ) compared with group I (control). In group IV (PHZ ± Vit C), these parameters and sperm DNA damage (*p* < 0.001) improved significantly when compared with PHZ-treated mice. Furthermore, PHZ caused a significant (*p* < 0.001) decrease in the fertilization rate and the percentage of pre-implantation embryos' (two cell embryo and blastocyst) formation in comparison to group I (control), and Vit C supplementation in mice of group IV improved significantly the fertilization rate (*p* = 0.002), but it could not improve the percentage of two cell embryos and blastocyst production.

**Conclusion:**

The data from this study indicated that Vit C decreased the adverse effects of PHZ on the quality of sperm parameters and in vitro fertilization rate, but it is insufficient to restore the in-vitro embryonic development and fertility potential.

## 1. Introduction

Anemia is a common and serious world public health affecting people of all ages and is related to a high risk of morbidity and mortality, especially in developing countries (1). Exposure to some chemicals and drugs have been associated with red blood cell (RBC) destruction and causing hemolytic anemia. Phenylhydrazine (PHZ), due to its toxic effects on RBC, is a useful agent in experimental model study of hemolytic anemia (2). This compound was first prescribed at the end of the nineteenth century as an antipyretic drug and is now well-known for its ability to RBC hemolysis in animals and humans inducing hemolytic anemia (3).

Results of a previous study indicated that PHZ adversely affected the male reproductive system in mice that suggested to induce oxidative damage. It was indicated that administration of PHZ in mice for 35 days caused decrease in the epididymal sperm parameters quality (motility, count, and morphology). It was also shown that PHZ through the formation of lipid peroxidation causes DNA damage in sperms (4). Amer and colleagues reported that PHZ elevates reactive oxygen species (ROS) and lipid peroxidation and alleviates glutathione (5). These effects may have been reversed following antioxidant administration.

Oxidative stress is a major cause of reproductive failure, and it has been proven that male germ cells may be prone to oxidative damage due to the high amount of polyunsaturated fatty acids and low capacity for DNA repair (6). Other investigators have indicated that damage in testicular tissue is associated with lipid peroxidation and they suggested that high production of ROS may play a major role in inducing testicular degeneration, resulting in infertility (7). Thus, administration of compounds with antioxidant properties can help the body to combat against pathological status caused by ROS or free radicals.

Further studies have shown that antioxidants scavenge ROS are produced by leukocytes and protect sperm DNA from fragmentation and can also improve semen quality in persons exposed to oxidative stress (4, 8). Vitamin C (Vit C) or ascorbic acid is one of the major antioxidant compounds in biological systems (9). Supplementation of Vit C can protect the blood-testis barrier destruction induced by cadmium through the inhibition of oxidative stress (10). Talebi and colleagues indicated that Vit C as a potent antioxidant alleviates adverse effects of diabetes on the sperm parameters, chromatin maturity and apoptosis in mice (11). Vit C deficiency in guinea pigs caused great degeneration of the seminiferous tubules and reduced the weight of the reproductive system, including testis and accessory sex organs, and Vit C supplementation reversed this reduction (12).

Considering the positive effects of Vit C on the male reproductive system, the current study was conducted to evaluate the protective effects of Vit C on sperm parameters and IVF potential (embryonic development) in male mice exposed to PHZ-induced hemolytic anemia.

## 2. Materials and Methods

### Animals and treatment 

Thirty-two adult male NMRI mice (25–27 gr) were maintained in standard animal house conditions (temperature 23 ± 2${}^{\circ }$C and 12 hr light/dark cycles). The animals were randomly assigned into four groups (*n* = 8): group I – control group, received intraperitoneal (IP) normal saline (0.1 mL, daily); group II – PHZ group, received PHZ (Sigma-Aldrich, St. Louis, USA) at the dose of 8 mg/100 g/body weight/IP at first, followed later at the dose of 6 mg/100 g every 48 hr; group III – Vit C group, received Vit C (Shahre Darou Company, Tehran, Iran) daily at the dose of 10mg/kg/IP; and group IV – PHZ + Vit C group, received PHZ and Vit C at the same dose. The treatment time for all the groups was 35 days.

This duration of time of experiment was selected according to the timing of mouse spermatogenesis in previous studies (13, 14). The doses of PHZ and Vit C were selected according to previous studies (4, 11). During the study, the animals were accessed to food and water ad libitum. After 35 days, the mice were euthanized with an overdose of ketamine (Alfasan, Woerden, the Netherlands). Then, the caudal portion of both epididymides of each mouse was transferred and minced in a petri dish containing 1 mL of human tubal fluid medium (HTF; Sigma-Aldrich, St. Louis, USA) preheated at 37${}^{\circ }$C and then incubated for 30 min to release sperm from the epididymis.

### Sperm parameters analysis (Sperm count, motility, viability, and morphology)

After diluting, sperms were counted using a Neubauer slide under a light microscope. The motility of sperm was expressed by the percentage of motile sperm of the total sperm counted (15). For sperm viability, 20 µL of eosin solution was added into the same value of sperm sample and then 20 µL of nigrosine solution was added and smear prepared; after drying the slides, the percentage of unstained colorless alive sperms and red stained dead sperms were determined. The percentage of sperm morphology was evaluated using the slides stained by aniline blue (4). To determine all percentages (motility, viability, and morphology), 200 sperms were counted per slide.

### Aniline blue (AB) staining

AB stains lysine-rich histones and is considered as a marker of sperm chromatin evaluation used to detect sperm nucleus maturity. Briefly, dried smears from sperm solution were fixed for 30 min in 3% glutaraldehyde. The smears were stained with 5% AB for 7 min. In this test, normal mature sperms were pale and abnormal immature sperms were dark blue in color. Under a light microscope using 100× magnification, at least 200 spermatozoa were counted in each slide and the data were expressed as a percentage (4).

### Acridine orange (AO) staining

AO is a fluorescent staining that is used to evaluate chromatin integrity and determine damage to DNA. Indeed, this staining detects double- and single-stranded regions in sperm chromatin. Briefly, air-dried sperm smear slides were fixed for 2 h in Carnoy's fixative (methanol/acetic acid 1:3). Then, the slides dried at room temperature were stained with AO solution for 7 min and dried again. The slides were examined using a fluorescent microscope with 100× magnification and at least 200 spermatozoa per mouse were evaluated, and the percentage of normal sperms (green color) and abnormal ones (red color) were determined (4).

### In Vitro Fertilization (IVF)

#### Animals, oocytes collection and fertilization

At the end of the study (35 days), the male mice were prepared for IVF. The authors used 60 adult female (10 wk. old) mice to obtain enough oocytes for IVF assay. To induce superovulation and collect mature oocytes from oviducts, each female was intraperitoneal injected with 10 IU pregnant mare`s serum gonadotropin hormone (PMSG, Folligon, The Netherland) and followed after 48 hr with IP injecting of 10 IU human chorionic gonadotropin hormone (hCG). About 12–14 hr after hCG injection, female mice were sacrificed and then the fallopian tubes were removed and transferred to a drop of HTF-BSA medium previously equilibrated in an incubator (5% CO${}_{2}$, 37${}^{\circ }$C). The oviducts were dissected and the oocytes were removed, and after washing, were placed in droplets under mineral oil for fertilization. Then, 1×10${}^{6}$ capacitated sperms from each male mouse were added to oocytes in the fertilization droplets.

### Assessment of fertilization and embryonic development

**Figure 1:**
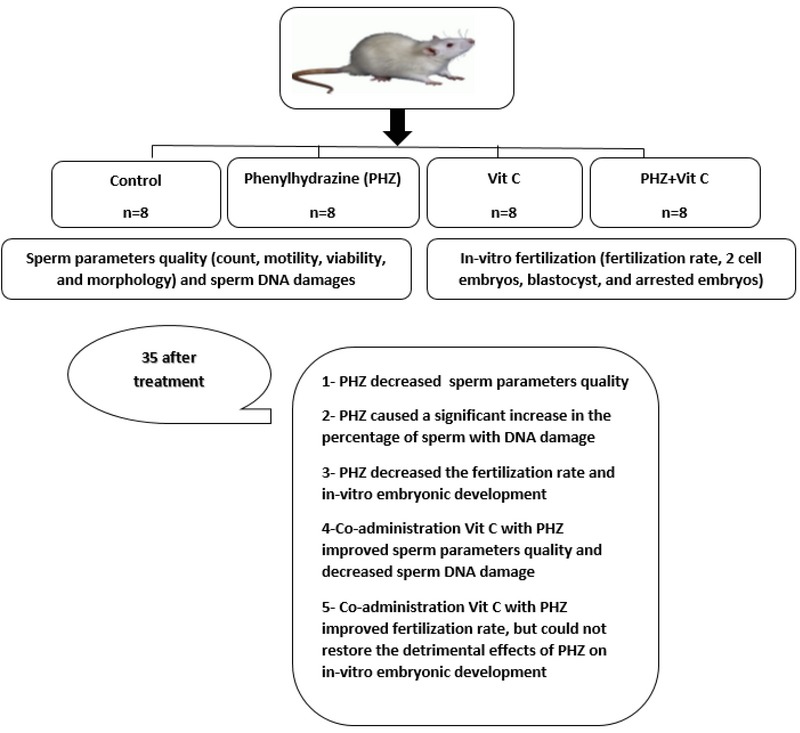
Flowchart for the experiment design.

Under an inverted microscope, the fertilization process was evaluated after 3–5 hr by observing two pronuclei. After culturing these zygotes for 24 hr, the number of two cell embryos were counted, and finally after 120 hr, the percentage of blastocyst development and arrested embryos were determined. The flowchart for the experiment design has been shown in Figure 1.

### Ethical consideration

This study was approved by the Ethics committee of Urmia University of Medical Sciences (IR.umsu.rec.1394.153).

### Statistical analysis

The data of IVF assay were analyzed by two proportional test using Minitab software (version 15.1, Minitab Inc., PA, USA). Other results were examined by One-Way ANOVA using SPSS 16 software (USA). All results were shown as means ± SD and a *p* < 0.05 was determined as statistically significant.

## 3. Results

### Sperm count and motility

Compared to group I (control) (35 × 10${}^{6}\pm$ 2.73), mice that received PHZ (17.12 × 10${}^{6}\pm$ 4.64) and PHZ + Vit C (26.12 × 10${}^{6}\pm$ 2.83) had lower epididymis sperm counts (*p* < 0.001 and *p* = 0.002, respectively). The results also showed that sperm counts in group IV (PHZ + Vit C) were significantly (*p* < 0.001) higher than group II (PHZ) (Table I). There was no significant difference between group III (Vit C) and group I. The percentage of sperm motility in studying groups are also shown in Table I. In comparison with group I (control), sperm motility showed a significant decrease in groups II (PHZ) and IV (PHZ + Vit C) (*p* < 0.001 and *p* = 0.008, respectively). In addition, the sperm motility of the mice receiving PHZ + Vit C was significantly (*p* = 0.007) greater than those of the mice receiving only PHZ.

### Sperm viability

The comparison of the percentage of alive sperm in group II (PHZ) with group I (control) indicated that it was significantly (*p* < 0.001) reduced. This decrease of sperm viability was significantly (*p* < 0.001) improved in group IV (PHZ + Vit C), but it was still significantly (*p* = 0.023) lower than group I (control). However, sperm viability in group IV (PHZ + Vit C) was significantly (*p* =0.006) greater than group II (PHZ) (Table I).

### Sperm morphology

Table I also shows the analysis of sperm morphology data. Compared to group I (control), the percentages of sperm with normal morphology in groups II (PHZ) and IV (PHZ + Vit C) were significantly low (*p* < 0.001). However, supplementation with Vit C in group IV (PHZ + Vit C) improved it significantly (*p* = 0.02) compared to group II (PHZ).

### Sperm DNA damage

The percentage of sperm with DNA damage in group II (PHZ) was significantly higher than groups I (control) and III (Vit C) (*p* < 0.001). A statistically significant reduction (*p* < 0.001) in the percentage of sperm with DNA damage was observed in group IV (PHZ + Vit C) when compared to group II (PHZ), but in comparison with group I, it was still higher (*p* < 0.001) (Table II).

### Sperm nucleus immaturity

Table II shows the results of analysis of sperm nucleus immaturity. The percentages of immature sperm in groups II (21.5 ± 4.79) and IV (9 ± 3.9) were significantly (*p* < 0.001) higher than groups I and III (2 ± 0.81 and 1.5 ± 0.57, respectively). However, in group IV (PHZ + Vit C), it was significantly (*p* < 0.001) reduced in comparison with group II (PHZ). Therefore, treatment with Vit C could not restore the mean percentage of immature sperms to control and Vit C groups levels.

### IVF assessments

Table III shows IVF and embryo development outcomes in different groups. The results revealed that the percentage of fertilization in group II (PHZ) was significantly lower than groups I (control) and IV (PHZ + Vit C), (*p* < 0.001 and *p* = 0.002, respectively). The percentage of two cell embryos formation in group II compared to group I was significantly reduced (*p* <0.001) but, in comparison with group IV, it was not significant. Table III also shows the percentage of blastocyst production. The results showed a significant (*p* < 0.001) reduction in group II compared to group I, but in comparison with group IV, the reduction was not significant. Overall, the percentage of arrested embryos before the blastocyst formation in group II (PHZ) was significantly higher than groups I (control) and III (Vit C). This result in group IV (PHZ + Vit C) was lower than group II (PHZ), but it was not significant.

**Table 1:** The results of the sperm parameters analysis in different groups.


**Groups**	**Sperm count (×10${}^{6}$)**	**Motility (%)**	**Viability (%)**	**Normal morphology (%)**
Group I (Control)	35 ± 2.73${}^{ab}$	68 ± 2.16${}^{ab}$	70.25 ± 4.75${}^{ab}$	85 ± 3.36${}^{ab}$
Group II (PHZ)	17.12 ± 4.64	36.5 ± 6.85	40 ± 6.16	49 ± 4.96
Group III (Vit C)	37.25 ± 2.32	69 ± 6.05	74.25 ± 4.78	89.66 ± 4.16
Group IV (PHZ + Vit C)	26.12 ± 2.83${}^{c}$	49.75 ± 4.27${}^{c}$	57.25 ± 3.77${}^{c}$	58.25 ± 5.31${}^{d}$

Data presented as mean ± SD The results were analyzed using one-way ANOVA

${}^{a}$Significantly different from PHZ group (*p* < 0.001)

${}^{b}$Significantly different from PHZ + Vit C (*p* < 0.01)

${}^{c}$Significantly different from PHZ group (*p* < 0.01)

${}^{d}$Significantly different from PHZ group (*p* < 0.05)

**Table 2:** The results of sperm DNA damage and chromatin immature in different groups.


**Groups**	**Sperm DNA damage (%)**	**Immature sperm (%)**
Group I (Control)	1.5 ± 0.57${}^{ab}$	2 ± 0.81${}^{ab}$
Group II (PHZ)	26.75 ± 4.03	21.5 ± 4.79
Group III (Vit C)	1.25 ± 0.5	1.5 ± 0.57
Group IV (PHZ + Vit C)	10.5 ± 3.8${}^{c}$	9 ± 3.9${}^{c}$

Data presented as mean ± SD

The results were analyzed using one-way ANOVA

${}^{a}$Significantly different from PHZ group (*p* < 0.001)

${}^{b}$Significantly different from PHZ + Vit C group (*p* < 0.001)

${}^{c}$Significantly different from PHZ group (*p* < 0.001)

**Table 3:** The outcomes of IVF in mice.


**Groups**	**Oocytes**	**Fertilized oocytes (%)**	**Two cell (%)**	**Blastocysts (%)**	**Arrested embryos (%)**
Group I (Control)	162	149 (91.97)${}^{ab}$	128 (85.97)${}^{ad}$	92 (61.74)${}^{ad}$	37 (38.25)${}^{ad}$
Group II (PHZ)	170	113 (66.47)	72 (63.71)	36 (33.96)	77 (68.14)
Group III (Vit C)	114	106 (92.98)	92 (86.79)	67 (63.21)	39 (36.79)
Group IV (PHZ + Vit C)	148	121 (81.75)${}^{c}$	78 (64.46)	46 (38.01)	75 (61.98)

Data presented as mean ± SD

The data were analyzed by 2 proportional test

${}^{a}$Significantly different from PHZ group (*p* < 0.001)

${}^{b}$Significantly different from PHZ + Vit C group (*p* < 0.01)

${}^{c}$Significantly different from PHZ group (*p* < 0.001)

${}^{d}$Significantly different from PHZ + Vit C group (*p* < 0.001)

## 4. Discussion

The data of this study showed that almost all sperm parameters, including count, motility and abnormal morphology, had a significant decline following PHZ administration, and that Vit C as an important antioxidant compound for male reproduction (16) improved them. The authors' findings also revealed that PHZ administration had a detrimental effect on IVF outcomes and embryonic development, and treatment with Vit C reverses only fertilization rate but is insufficient to restore in vitro embryonic growth.

Recent reports have indicated that infertility is considered to be a major clinical problem, and it has been reported in one in four couples (17, 18). Oxidative stress is one of the main causes of infertility in men (19). Overproduction of ROS in sperm induces nuclear DNA fragmentation, lipid peroxidation that leads to cell death (20, 21). Due to the high amount of polyunsaturated fatty acids in the spermatozoa and also the lack of ability for DNA repair, they are sensitive to ROS elevation. Thus, the increased ROS amount has been associated with declined sperm parameters quality and quantity (6, 20).

In agreement with our findings, Mozafari and colleagues showed that sperm parameters' quality and quantity significantly decreased in mice treated with PHZ compared to control mice. They showed that PHZ increased the level of malondialdehyde (MDA) in testicular tissue in mice. MDA is an indicator of the degree of lipid peroxidation and causes testis tissue damages (4).

Prior studies also demonstrated that short-term administration of rats with PHZ resulted in hemolysis anemia with decreased RBC and hemoglobin (Hb) (22, 23). Hemolysis or declined levels of RBC induced by PHZ leads to overproduction of ROS and lipid peroxidation. Thus, oxidative damage plays a major role in the pathogenesis of PHZ-induced testicular damage. To protect sperm from ROS, it is important to use antioxidative defense mechanisms in the testis (24). Considering the effect of PHZ on the antioxidant state, the authors hypothesized that Vit C as a potent antioxidant could prevent the PHZ-dependent damages in mice sperm parameters and fertility potential.

These findings revealed that PHZ significantly reduced the sperm count and motility. The decrease of sperm count is largely related to the onset of testicular damage, and reduction in sperm motility is mainly dependent on oxidative stress damage. Thus, a decrease in sperm motility is the first sign of elevated ROS level (25, 26). This increase in ROS level affects sperm enzymatic content and enhances phospholipids peroxidation, which eventually leads to a decrease in cell membrane fluidity and sperm motility (27). However, supplementation with Vit C resulted in a notable improvement in sperm count and motility in mice received PHZ. Observations have indicated that PHZ-treated mice showed a significant increase in the percentage of sperm with abnormal morphology. In contrast to our findings, in an experimental study in diabetic rats, it was indicated that treatment with Vit C reverses only testicular damage but is not able to restore sperm motility and fertility (28).

In order to find out how PHZ induce sperm parameters impairment, it should be considered that during spermatogenesis process a part of sperm cytoplasm must be phagocytosis and removed by Sertoli cells (29, 30). Remaining of these cytoplasmic droplets in the middle piece of sperm impair sperm maturation. Therefore, it is logical to hypothesize that the adverse effects of PHZ on sperm maturation, during spermatogenesis, exert partly through impairing the normal physiological function of Sertoli cells, which may happen due to PHZ-induced oxidative stress damage. On the other hand, Vit C-treated mice revealed a significant reduction in the percentage of abnormal sperm. Therefore, it can be postulated that Vit C recovers Sertoli cells physiologic activity by improving the antioxidant state. These findings in PHZ-induced low sperm count were in accordance with a previous study (4). This reduction in sperm count may also be associated with a decrease in production of testosterone hormone due to damage to Leydig and Sertoli cells following PHZ supplementation (4).

Regarding the AO staining test, which can detect abnormal single-stranded DNA from normal double-stranded sperms, it can be assumed that PHZ elevates denaturation of sperm DNA strands and co-treatment with Vit C reduces sperm DNA damage induced by PHZ, probably through a reduction in ROS production. In accordance with the current results, in a prior study, it was shown that the addition of Vit C to semen and prepared spermatozoa improved sperm parameters quality and DNA integrity following verification (31).

About AB test, it should be considered that there are some different reports on the use of vitamins to improve sperm chromatin abnormalities. Silver and colleagues demonstrated that the administration of Vit C and E did not have any positive effects on sperm chromatin maturity or condensation (32). This controversy may be due to the different mechanisms of infertility in the studies and the administered doses.

In vitro fertilization and embryonic development in the PHZ-treated group was significantly lower than the control group and the percentage of embryonic arresting was also higher. Studies indicated that there is a positive correlation between sperm parameters quality and fertilization rate, embryonic growth and high incidence of birth defects both in vivo and in vitro (8, 33, 34). In the present study, Vit C was only able to ameliorate the side effect of PHZ on fertilization rate. Thus, the findings of the present study showed that Vit C supplementation could not improve the percentage of two cell embryos, blastocyst and embryo arresting in mice co-treated with PHZ. Considering these findings, it can be postulated that Vit C significantly but not completely protects against the PHZ-induced testicular damage. The present study is the first report on the relationship between sperm parameters and embryonic development impairments and Vit C supplementation in PHZ-induced male reproductive system disorders. Thus, to compare these findings with other studies, the authors did not find a similar study. However, they were not able to explain why Vit C can only improve the fertilization rate. This may be because the Vit C dose used in this study was not sufficient to recover the embryonic development completely.

The testis is sensitive to different stressors including hyperthermia, inflammation, radiation and also exposure to toxic agents that induce apoptosis of germ cells. Sertoli cells in testicular tissue have the main role in supporting normal spermatogenesis by creating a special environment that induces germ cells differentiation and also controls the entry of nutrients, hormones and other agents into seminiferous tubules (35). Thus, Sertoli cells are important in creating blood-testis-barrier and normal physiologic function of the male reproductive system is dependent on the maintenance of this selective physiologic barrier (35).

The mechanism by which Vit C enters the adluminal compartment of seminiferous tubules to enter the developing germ cells was an unsolved problem for many years. So it is obvious that for Vit C to reach the adluminal part of seminiferous tubules, it must pass through Sertoli cells by passive diffusion or facilitative transporters (36). Angulo and colleagues explained this problem by showing that Sertoli cells express two functional active Vit C transporters that give the ability to Sertoli cells to metabolize and regulate the delivery of Vit C to germ cells. Thus, Sertoli cells have a major role in controlling Vit C concentration in the adluminal part of seminiferous tubules (37).

## 5. Conclusion

The data obtained from this study suggest that Vit C as a potent antioxidant can attenuate the detrimental effects of PHZ on sperm parameters and DNA integrity and improve in vitro fertilization rate but is insufficient to restore the in vitro embryonic development and fertility potential.

##  Conflict of Interest

There are no conflicts of interest to declare.
